# Supine versus prone position in percutaneous nephrolithotomy: a systematic review and meta-analysis

**DOI:** 10.12688/f1000research.22940.1

**Published:** 2020-04-02

**Authors:** Ponco Birowo, William Tendi, Indah S. Widyahening, Nur Rasyid, Widi Atmoko

**Affiliations:** 1 Department Urology, Faculty of Medicine Universitas Indonesia / Cipto Mangunkusumo Hospital, Jakarta Pusat, DKI Jakarta, 10430, Indonesia; 2 Department of Community Medicine, Faculty of Medicine Universitas Indonesia, Jakarta Pusat, DKI Jakarta, 10430, Indonesia

**Keywords:** Complication rate, Percutaneous nephrolithotomy, Prone, Stone free rate, Supine

## Abstract

**Background: **The decision for using supine or prone position in percutaneous nephrolithotomy (PCNL) is still debatable. The aim of this study is to compare the efficacy and safety profile of the supine and prone position when performing PCNL.

**Methods: **A systematic electronic search was performed using the database from MEDLINE, Cochrane library and Google Scholar from January 2009 to November 2019. The outcomes assessed were stone free rate, major complication rate, length of hospital stay and mean operation time.

**Results: **A total of 11 articles were included in qualitative and quantitative analysis. The efficacy of PCNL in supine position as determined by stone free rate is significantly lower than in prone position (OR: 0.74; 95% CI: 0.66 – 0.83; p<0.00001), However, major complication rate is also lower in the supine group compared with the prone group (OR: 0.70; 95% CI: 0.51 – 0.96; p=0.03). There is no statistically significant difference in the length of hospital stay and mean operation time between both groups.

**Conclusion: **Prone position leads to a higher stone free rate, but also a higher rate of major complication. Thus, the decision of using which position during PCNL should be based on the surgeon’s experience and clinical aspects of the patients.

## Introduction

Nephrolithiasis is one of the most common urological diseases worldwide. It is defined as a condition where mineral deposits are found in the kidney, either free in the renal calyces and pelvis or attached on the renal papillae ^1^. The prevalence is varied between regions, ranging between 7–13% in North America, 5–9% in Europe, and 1–5% in Asia ^2^. The most common stone composition is calcium, comprising about 80% of all urolithiasis ^3^.

Depending on stone burden, the treatment of nephrolithiasis also has a wide range of options. Active management includes extracorporeal shockwave lithotripsy (ESWL), retrieval by ureteroscopy (URS), and percutaneous nephrolithotomy (PCNL). The current guideline generally recommends ESWL for smaller stones (up to 20 mm) and PCNL for larger stones (>20 mm) regardless of the location inside the kidney ^4^.

While PCNL has higher free stone rates with a similar recurrence and complication rate compared with ESWL, this procedure also has its own preparation including a guiding system, anesthesia, and positioning of the patient. The conventional position of PCNL is prone, which allows direct access to the posterior calyx with minimal risk of bowel puncture. However, this positioning method limits the possibility of switching anesthesia from regional to general. The alternative position is supine, which allows general anesthesia switching and combination technique of antegrade and retrograde approaches. Moreover, this position is also more preferred in patients with cardiac comorbidity. However, working space and the possibility of multiple channels are limited ^5^. The aim of this study is to determine whether one position is more superior than the other, by comparing efficacy and safety profiles using a systematic review and meta-analysis approach.

## Methods

### Description of condition and intervention

The target population in this study is patients with renal stone of 20 mm or more in size who underwent PCNL. The intervention to the patients is PCNL in prone position, compared with PCNL in supine position. Prone is a classic position in PCNL procedure, described in 1976 when PCNL was first introduced. The original prone position consists of a two-stage procedure. The first stage is in supine position, where anesthesia is given and retrograde access to the upper urinary tract is established. The patient is then repositioned to a prone position, and supports are placed under the thorax and upper abdomen. All pressure points are also padded ^6^.

In contrast, a supine prone only needs one stage, in which the patient is placed supine with ipsilateral flank held up with a 3-liter saline bag. This original position was first introduced by Valdivia-Uria *et al.* and has been modified over time ^7^. One popular modification of Valdivia position is the Galdakao modification. This position is slightly more lateral; the contralateral leg of the patient is flexed and abducted, while the ipsilateral leg is extended. A 3-liter bag is also placed to raise the flank ^6^.

Apart from the Valdivia position and its modifications, a complete supine position was also introduced by Falahatkar *et al.* ^8^ This position does not require an elevation of the flank. The patient is simply put in a supine position at the edge of the table, with legs extended. The patient’s arms are stretched, abducted and supported.

The outcome of this study is the efficacy of both positions, determined by stone free rate and safety profile, determined by the occurrence of major complications.

### Database searching and literature screening

A systematic search was carried out with the date last searched in 14 February 2020, using the database from MEDLINE, with keywords of “(((supine[Title/Abstract]) AND prone[Title/Abstract])) AND ((PCNL[Title/Abstract]) OR percutaneous nephrolithotomy[Title/Abstract])”, and Cochrane library, with keywords of “prone in Title Abstract Keyword AND supine in Title Abstract Keyword AND PCNL in Title Abstract Keyword”, and Google Scholar with keywords of “prone AND supine AND percutaneous nephrolithotomy”. After we identified the articles, we removed the duplicates and further screened the articles. The reporting is based on Preferred Reporting Items for Systematic Reviews and Meta-Analyses (PRISMA) algorithm.

### Study selection

Two reviewers (PB and WT) independently appraised the articles, and a discussion was conducted when disagreement occurred. The relevance of the articles is determined by reading through the titles and abstracts. The inclusion criterion is a comparative study between the supine and prone position in PCNL procedure, and the articles were written in English. The exclusion criteria are non-comparative studies, studies that combine PCNL with other techniques of stone extraction such as URS or retrograde intrarenal surgery, not focused on comparing supine and prone position in PCNL, and inclusion of confounding factors such as a difference in guiding method when performing PCNL in each position, since this difference will lead to intervention bias. The quality of each article included were then tested using Jadad scale for randomized controlled trials (RCT) and Newcastle-Ottawa scale for non-RCTs ^9, 10^.

### Data extraction

Data extraction from the articles was performed by two authors (NR and WA), and any disagreement was settled by consensus. The variables extracted from the articles included the first author’s name, year of publication, stone free rate, percentage of major complications, length of hospital stay, and mean operation time. Stone free condition is defined as the absence of residual fragments of ≤ 4 mm after procedure. Major complications are defined as those with a Clavien score of III or more ^11^.

### Statistical analysis

Meta-analysis was performed by Review Manager 5.3. The results were described as odds ratio (OR) with 95% confidence interval (CI) for dichotomous variables, and as a mean difference with 95% CI for continuous variables. Heterogeneity was analyzed using a Chi square and I ^2^ test. The data was analyzed using the random-effect model when I ^2^ >25%, and fixed-effect model when I ^2^ is less than 25%. The analysis is considered statistically significant when p value is less than 0.05. For studies that provided the minimum and maximum value instead of standard deviation (SD) for the mean difference analysis, estimated SD were calculated with the formula derived from a study by Walter and Yao (2007) ^12^. In addition, for studies that provided 95% Confidence Interval (CI) instead of SD, the value of SD was calculated using the formula described in the Cochrane Handbook ^13^.

## Results

### Literature search

**Figure 1.**
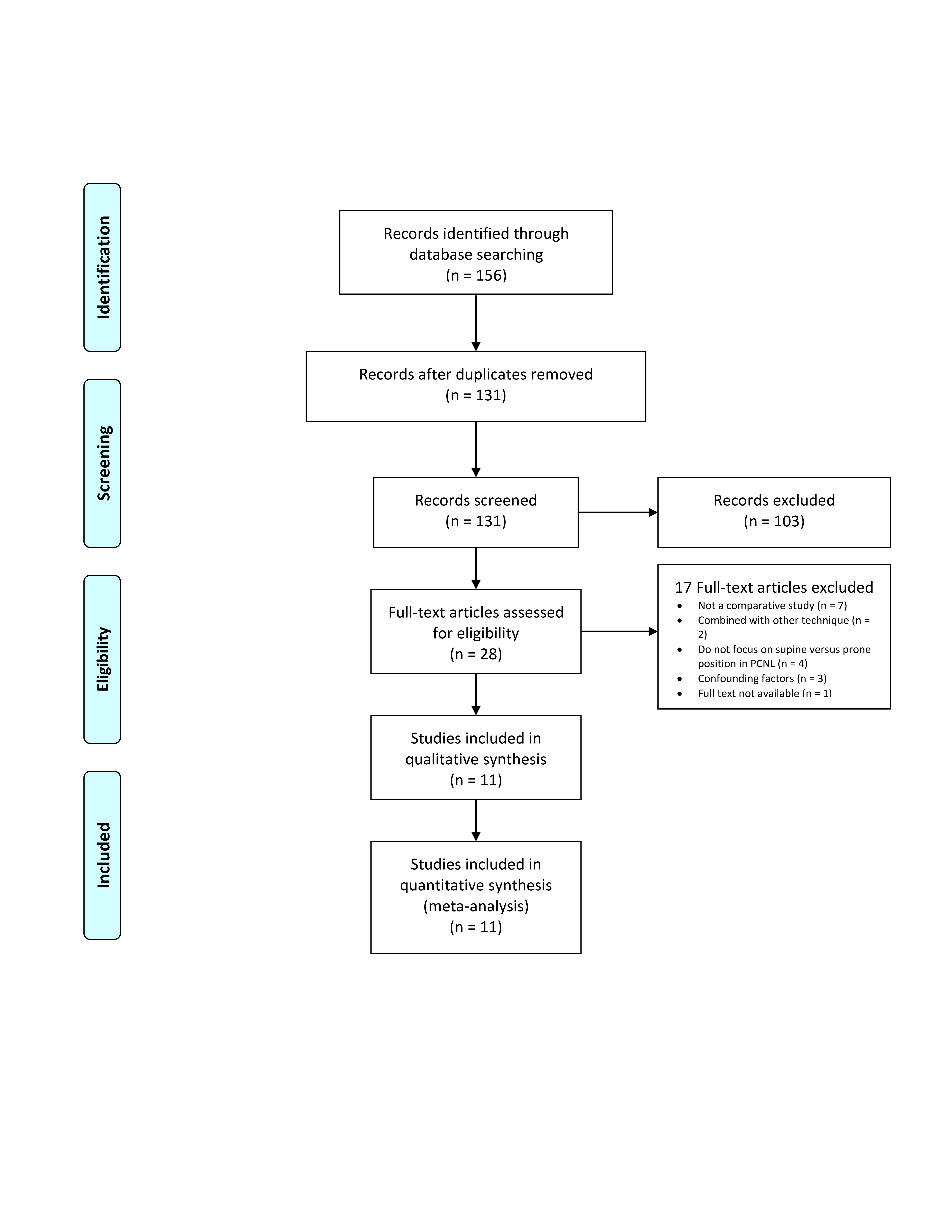
PRISMA method of article screening.

Following the result of article screening and the application of exclusion criteria, a total of 156 articles were found from the three databases. After removing duplicates, a total of 131 studies were screened for relevance, of which only 11 articles were included in qualitative and quantitative analysis ( Figure 1).

### Study characteristics

Quality assessment of the articles is shown in Table 1. Study characteristics, including the study design, mean age, and stone burden, is shown in Table 2.

**Table 1.** Quality assessment of the articles included. RCT, randomized controlled trial.

Articles	Study design	Quality assessment	
		Jadad scale	Newcastle- Ottawa scale
Melo PAS, *et al.* (2019) ^8^	Cohort	-	8
Gokce MI, *et al.* (2017) ^14^	Cohort	-	8
Mahmoud M, *et al.* (2017) ^15^	RCT	2	-
Wood GJA, *et al.* (2017) ^16^	Cohort	-	7
Astroza G, *et al.* (2013) ^17^	Cohort	-	6
Kan RW, *et al.* (2013) ^18^	Cohort	-	8
Karami H, *et al.* (2013) ^19^	RCT	1	-
Sanguedolce F, *et al.* (2013) ^20^	Cohort	-	6
Arrabal-Martin M, *et al.* (2012) ^21^	Cohort	-	7
Wang Y, *et al.* (2012) ^22^	Cohort	-	8
Valdivia JG, *et al.* (2011) ^7^	Cohort	-	8

**Table 2.** Characteristics of studies included.

Articles	Cases (n)		Mean age (years)		Stone size (mm/mm ^2^)	
	Supine	Prone	Supine	Prone	Supine	Prone
Melo PAS, *et al.* (2019)	294	99	49.14	47.66	29.76	30.34
Gokce MI, *et al.* (2017)	39	48	47.5	49.2	47.3	45.6
Mahmoud M, *et al.* (2017)	20	20	42.35	41.15	27.1	25.7
Wood GJA, *et al.* (2017)	28	104	45.89	44.98	-	-
Astroza G, *et al.* (2013)	232	1079	51.8	49.8	-	-
Kan RW, *et al.* (2013)	25	35	67	63	36.9	44.8
Karami H, *et al.* (2013)	50	50	44.4	41.5	28.2	28.3
Sanguedolce F, *et al.* (2013)	65	52	53	49	20.6	18.1
Arrabal-Martin M, *et al.* (2012)	24	32	49	47	510	530
Wang Y, *et al.* (2012)	6	12	44.8	43.8	36	33
Valdivia JG, *et al.* (2011)	1138	4637	51	48.8	470.6	449.1
**Total patients**	**1921**	**6168**	-	-	-	-

### Stone free rate

**Figure 2.**
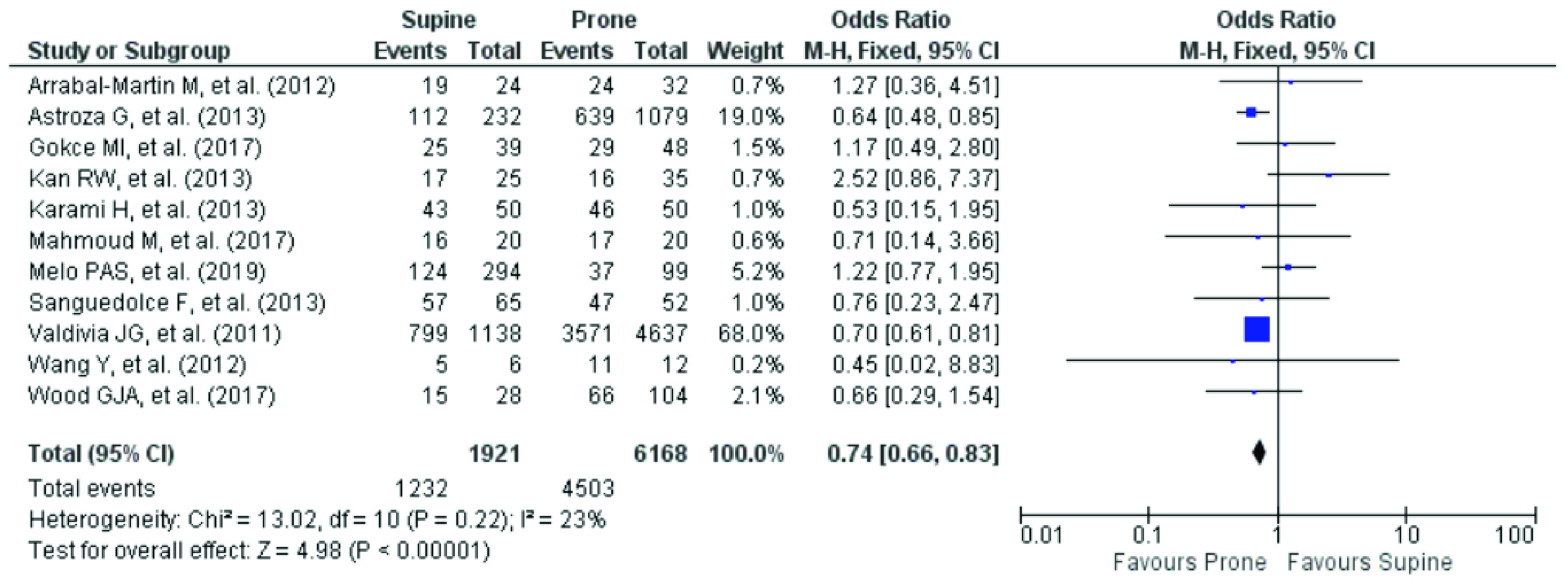
Forest plot comparing stone free rate in prone and supine groups.

All 11 studies reported the stone free rate of both supine and prone groups. A meta-analysis of these studies showed that there was a statistically significant lower stone free rate in the supine group (OR: 0.74; 95% CI: 0.66 – 0.83; p<0.00001; Figure 2).

### Major complication rate

**Figure 3.**
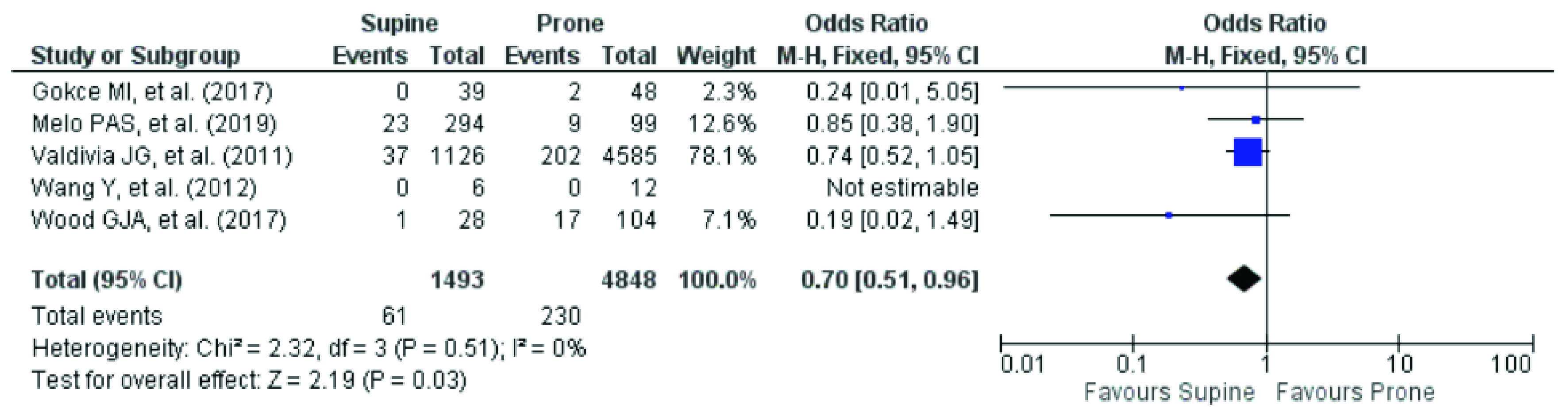
Forest plot comparing major complication rate in prone and supine groups.

Major complication rate is defined as Clavien score of 3 of more in this study. There were only 5 articles that reported the complication rate using Clavien score. Figure 3 showed that there is a statistically significant lower complication rate in the supine group (OR: 0.70; 95% CI: 0.51 – 0.96; p=0.03).

### Length of hospital stay

**Figure 4.**
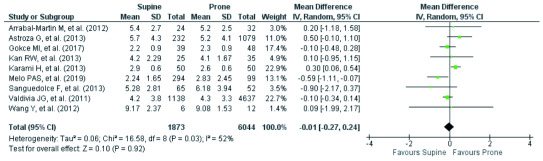
Forest plot comparing length of hospital stay in prone and supine groups.

There were nine studies that reported mean days of hospital stay in both groups. The forest plot in Figure 4 shows that there is no difference in the length of hospital stay between groups (Mean difference: -0.01; 95% CI: -0.27 – 0.24; p=0.92).

### Mean operation time

**Figure 5.**
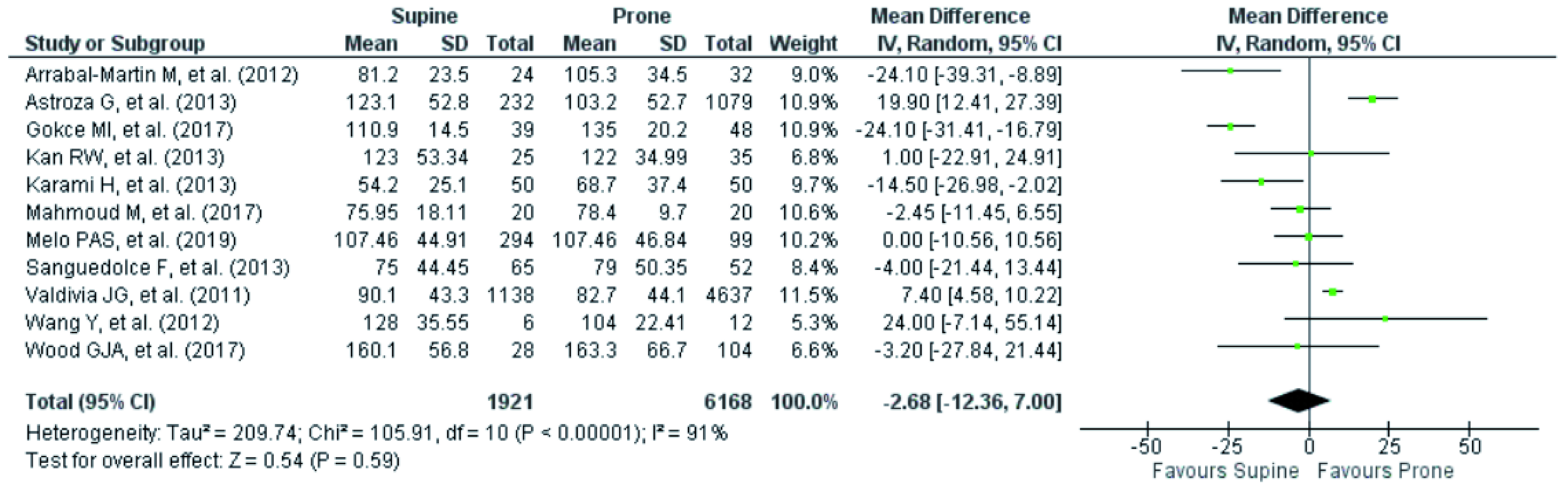
Forest plot comparing mean operation time in prone and supine groups.

Mean operation time was reported in all studies. The meta-analysis in this parameter showed that there is no difference in mean operation time between these two groups (Mean difference: -2.68; 95% CI: -12.36 – 7.00; p=0.59; Figure 5).

## Discussion

According to our review, supine and prone position during PCNL share a similar mean operation time and duration of hospital stay. This result is important so that the surgeons will be able to confidently decide the position based on their experiences and the patient’s comorbidities. In addition, the study conducted by Melo *et al.* showed similarities between both positions in terms of the number of puncture tracts, blood transfusion rate, and mean drop in hemoglobin level ^11^.

However, despite the similarities, this study also found significant differences between these two groups. The authors choose stone free rate and major complication as the main outcome of this article to help identify which position is safe in PCNL and whether there is a difference in the efficacy. Interestingly, both of these outcomes were statistically different.

Our study found that the supine position had a lower major complication rate than prone position. Literature revealed that the original (Valdivia) position is reportedly safe, and endoscopic instruments can be moved more freely because the puncture site of the abdominal wall is performed more laterally and away from the lumbar muscles. The tract in this position also preserves a low pressure in the renal pelvis, reducing the risk of fluid absorption. Moreover, risk of colonic puncture might be reduced because the bowel is not pressed towards the kidney. Should a rigid ureteroscopy be needed simultaneously with PCNL, a modified Valdivia position can be performed by flexing and supporting the patient’s ipsilateral leg, and the contralateral leg descended. The supine position also has the advantage of easier management of cardiac and respiratory emergencies ^6^.

Moreover, the Galdakao-modified position allows more instrument manipulation than the original supine position. Furthermore, it also enables simultaneous retrograde access to the kidney and there is no need to reposition thus the asepsis and antisepsis procedure needs to be performed only once ^6^.

In the complete supine position, the lack of flank support allows more feasible access to the upper pole of the kidney because there is no risk of cephalad sliding of the kidney, as observed in the supine position with flank support ^6^. The supine position also has the advantages of easier access to the upper pole after lower pole puncture ^23^.

However, it should also be noted that while there are many advantages to the supine position, the flank in this position is not fully exposed, therefore reducing the possibility of multiple access when needed. In addition, the state of low compressed abdomen allows the kidney to move more freely, making the navigation of the instrument towards the kidney more challenging, and the chance of failed access is higher ^6, 7, 16^.

Additionally, our study found that stone free rate was significantly higher in prone position. The major advantage in this position is the fully exposed lumbar area. This allows a possibility of several puncture sites, and easier access to the upper pole kidney. Moreover, the working area is greater, providing enough space for instrument manipulation ^6^.

However, the two-stage nature of this position usually prolongs the operating time, and a prone position makes it difficult for the anesthetists to attend cardio-respiratory emergency. The risk of ocular complications has also been described because of the increase in intra-ocular pressure ^6^.

The limitation in our study is that the number of articles providing data of major complication rate in terms of Clavien score was limited and there were too many heterogeneities in the length of hospital stay and mean operation time variables. Therefore, the authors believe that another comprehensive study should be performed in urology centers in which the surgeons excel in both supine and prone position when performing PCNL and have a larger sample size.

The implication of this study is that it exposed the benefit and disadvantages of both supine and prone position, which in turn can be used as a decision guide for clinicians who want to perform PCNL.

## Conclusion

In conclusion, the prone position leads to a higher stone free rate than supine position. However, in terms of safety profile, supine position provides a better choice than the prone position. There is no difference in both the length of hospital stay and mean operation time between prone and supine position. Therefore, it can be inferred that there is no position that has absolute superiority and it is important to note that both supine and prone position in PCNL procedure have their respective advantages and disadvantages. Thus, the decision of choosing the position when performing PCNL should be based on clinical status of the patient and the experience of the surgeon.

## Data availability

### Underlying data

All data underlying the results are available as part of the article and no additional source data are required.

### Reporting guidelines

Open Science Framework: PRISMA checklist for article ‘Supine versus prone position in percutaneous nephrolithotomy: a systematic review and meta-analysis’, https://doi.org/10.17605/OSF.IO/23GND ^24^.

Data are available under the terms of the Creative Commons Zero "No rights reserved" data waiver (CC0 1.0 Public domain dedication).
