# Supplementary material for: Supine versus prone position in percutaneous nephrolithotomy: a systematic review and meta-analysis
Source: F1000Res. 2020 Sep 15;9:231. Originally published 2020 Apr 2. [Version 3] doi: 10.12688/f1000research.22940.3 (PMC7509599; doi:10.12688/f1000research.22940.3)
Supplement: Peer review history

# Peer review: Reviewer response for version 3

**DOI:** 10.5256/f1000research.29538.r71389

**Published:** 2020-10-05

**Authors:** Noor Buchholz

**Affiliations:** 1 U-merge Ltd. (Urology for Emerging Countries), London, UK

Are the rationale for, and objectives of, the Systematic Review clearly stated?

Yes

Is the statistical analysis and its interpretation appropriate?

Yes

Are sufficient details of the methods and analysis provided to allow replication by others?

Yes

Are the conclusions drawn adequately supported by the results presented in the review?

Yes

Reviewer Expertise:

NA

I confirm that I have read this submission and believe that I have an appropriate level of expertise to confirm that it is of an acceptable scientific standard.

# Peer review: Reviewer response for version 3

**DOI:** 10.5256/f1000research.29538.r71388

**Published:** 2020-09-22

**Authors:** Mohammed S. ElSheemy

**Affiliations:** 1 Urology department, Kasr Al-Ainy Hospitals, Cairo University, Cairo, Egypt

The authors responded to all queries and the manuscript is ready now to be accepted.

Are the rationale for, and objectives of, the Systematic Review clearly stated?

Yes

Is the statistical analysis and its interpretation appropriate?

I cannot comment. A qualified statistician is required.

Are sufficient details of the methods and analysis provided to allow replication by others?

No

Are the conclusions drawn adequately supported by the results presented in the review?

Yes

Reviewer Expertise:

Urolithiasis, endourology, PCNL, Mini-PCNL, URS, SWL

I confirm that I have read this submission and believe that I have an appropriate level of expertise to confirm that it is of an acceptable scientific standard.

# Peer review: Reviewer response for version 2

**DOI:** 10.5256/f1000research.28883.r70049

**Published:** 2020-08-26

**Authors:** Mohammed S. ElSheemy

**Affiliations:** 1 Urology department, Kasr Al-Ainy Hospitals, Cairo University, Cairo, Egypt

The manuscript was improved markedly as the authors replied to most of the previous comments. However, there are still remaining points that need revision.

**Introduction**

- In the previous version, we commented as follows: [The authors reported that: [While PCNL has higher free stone rates with a similar recurrence and complication rate compared with ESWL,...]. This should be cited. Additionally, the complication rates are different between both procedures. Similarly, the recurrence rate is different.
  - The authors replied by putting citation which was a secondary source [reference 5: Ganpule AP, et al.: Percutaneous nephrolithotomy (PCNL) a critical review. *Int J Surg.* 2016;36(Pt D):660–664.] not the original source [B. Turna, et al. Management of calyceal diverticular stones with extracorporeal shock wave lithotripsy and percutaneous nephrolithotomy; long term outcome. BJU Int., 100 (2007), pp. 151-156]. The original source should be read by authors then cited instead of the secondary source especially that the original source is available online (free access).
  - Additionally, the original source was on stones in calyceal diverticula which is totally different than the scope of the current study and also the outcome of PCNL and SWL generally which is different than the outcome of the rare condition of stones in calyceal diverticula.
  - Furthermore, even in the original study; no one can comment on the recurrence rate as the SFR was 80% in PCNL compared to only 20% in SWL; subsequently, 80% of cases in SWL were already with residual stones preventing the use of the term (recurrence).
  - Moreover, the complications in the original study were totally different as blood transfusion and chest tube were required in some cases of PCNL while SWL had milder complications.
  - Furthermore, the original study was retrospective with a huge difference in stone size (mean; 20 mm (Max. 60 mm) for PCNL vs 11 mm in SWL (max; 30 mm))

**Methods**

- The authors added case-control studies in the inclusion criteria. However, they were not included in the metanalysis.

**Results**

- Our previous comment on Figure 1 was that the authors should clarify why 103 studies were excluded out of 131. The authors replied that the explanation was presented in the text but our comment was to present that also in the figure.
- It was great to respond by adding a separate analysis for the need for blood transfusion, sepsis, and visceral injuries complications including pleural injury. However, the authors should comment on each separate analysis before commenting on the combined analysis as there was no significant difference in each separate analysis.
- The authors have added more details to Table 2; however, there are still other necessary details missing:
  - The definition of SFR.
  - If SFR was calculated after one session or after 2nd look PCNL (this was noted in one study but unknown for remaining studies).
  - If any procedure was Mini-PCNL and the caliber of the used renal track (sheath).
  - If there were congenital renal anomalies.

**Discussion**:

- The discussion section requires more effort especially the initial 3-4 paragraphs. The introduction then subsequent points in the discussion section should be in a logical order.

Are the rationale for, and objectives of, the Systematic Review clearly stated?

Yes

Is the statistical analysis and its interpretation appropriate?

I cannot comment. A qualified statistician is required.

Are sufficient details of the methods and analysis provided to allow replication by others?

No

Are the conclusions drawn adequately supported by the results presented in the review?

Yes

Reviewer Expertise:

Urolithiasis, endourology, PCNL, Mini-PCNL, URS, SWL

I confirm that I have read this submission and believe that I have an appropriate level of expertise to confirm that it is of an acceptable scientific standard, however I have significant reservations, as outlined above.

# Response

**Published:** 2020-09-10

**Authors:** Ponco Birowo

**Affiliations:** Department of Urology Faculty of Medicine Universitas Indonesia/ CIpto Mangunkusumo Hospital, Indonesia

**Introduction**

 Reviewers’ comment:

1. In the previous version, we commented as follows: [The authors reported that: [While PCNL has higher free stone rates with a similar recurrence and complication rate compared with ESWL,...]. This should be cited. Additionally, the complication rates are different between both procedures. Similarly, the recurrence rate is different.
  1. The authors replied by putting citation which was a secondary source [reference 5: Ganpule AP, et al.: Percutaneous nephrolithotomy (PCNL) a critical review. Int J Surg. 2016;36(Pt D):660–664.] not the original source [B. Turna, et al. Management of calyceal diverticular stones with extracorporeal shock wave lithotripsy and percutaneous nephrolithotomy; long term outcome. BJU Int., 100 (2007), pp. 151-156]. The original source should be read by authors then cited instead of the secondary source especially that the original source is available online (free access).
  2. Additionally, the original source was on stones in calyceal diverticula which is totally different than the scope of the current study and also the outcome of PCNL and SWL generally which is different than the outcome of the rare condition of stones in calyceal diverticula.
  3. Furthermore, even in the original study; no one can comment on the recurrence rate as the SFR was 80% in PCNL compared to only 20% in SWL; subsequently, 80% of cases in SWL were already with residual stones preventing the use of the term (recurrence).
  4. Moreover, the complications in the original study were totally different as blood transfusion and chest tube were required in some cases of PCNL while SWL had milder complications.
  5. Furthermore, the original study was retrospective with a huge difference in stone size (mean; 20 mm (Max. 60 mm) for PCNL vs 11 mm in SWL (max; 30 mm))

Authors’ response:

1. Thank you very much for your kind response. We have revised the statement above and added the original citation as reference number 5 (Page 3, Introduction section, Paragraph 3).

**Methods**

 Reviewers’ comment:

1. The authors added case-control studies in the inclusion criteria. However, they were not included in the metanalysis.

Authors’ response:

1. Thank you for your kind response. We did not include the case control in our meta-analysis because we did not manage to find case control studies that fulfill our inclusion criteria or in accordance with our topic. Nevertheless, we added this sentence to the manuscript. (Page 5-6, Method section, subsection Study Characteristics, Paragraph 1).

**Results**

 Reviewers’ comment:

1. Our previous comment on Figure 1 was that the authors should clarify why 103 studies were excluded out of 131. The authors replied that the explanation was presented in the text but our comment was to present that also in the figure.
2. It was great to respond by adding a separate analysis for the need for blood transfusion, sepsis, and visceral injuries complications including pleural injury. However, the authors should comment on each separate analysis before commenting on the combined analysis as there was no significant difference in each separate analysis.
3. The authors have added more details to Table 2; however, there are still other necessary details missing:
  - The definition of SFR.
  - If SFR was calculated after one session or after 2nd look PCNL (this was noted in one study but unknown for remaining studies).
  - If any procedure was Mini-PCNL and the caliber of the used renal track (sheath).
  - If there were congenital renal anomalies.

Authors’ response:

1. Thank you for your recommendation, we have revised Figure 1 and put the explanation in the figure (Page 5, Figure 1). We have also updated the PRISMA flowchart in the Open Science Framework Database accordingly (Reference number 27).
2. Thank you for your kind comment, we have added separate comment regarding the result of the component of the subgroup analysis (Page 10, Result section, subsection Major Complication Rate, Paragraph 2).
3. Thank you for the suggestion, we have added the details required separately in the table 2 and table 3. (Page 7-9, Result section, Table 2 and Table 3).

**Discussion**

 Reviewers’ comment:

1. The discussion section requires more effort especially the initial 3-4 paragraphs. The introduction then subsequent points in the discussion section should be in a logical order.

Authors’ response:

1. Thank you for your kind comment. We have revised the discussion section, including adding and re-arranging the paragraph so that our conceptual of thinking in this study is in order and clearly described. (Page 11-14, Discussion section).

# Peer review: Reviewer response for version 1

**DOI:** 10.5256/f1000research.25326.r67732

**Published:** 2020-07-31

**Authors:** Mohammed S. ElSheemy

**Affiliations:** 1 Urology department, Kasr Al-Ainy Hospitals, Cairo University, Cairo, Egypt

The authors presented their meta-analyses comparing prone versus supine PCNL for the management of renal stones > 2 cm. They reported a higher SFR with prone technique but a better safety profile with supine technique. However, there are many points that require more details especially in the results and discussion section. The authors did not comment on studies heterogenousities or quality. Many points were not presented in each study (detailed in the comments on the results and discussion section). The authors did not discuss previous meta-analyses on the same issue nor clarifying the addition or difference in the current study.

**Introduction:**

- The authors reported that: [While PCNL has higher free stone rates with a similar recurrence and complication rate compared with ESWL,...]. This should be cited. Additionally, the complication rates are different between both procedures. Similarly, the recurrence rate is different.   

**Method:**

- The authors should clarify the age of patients and the design of the study (RCT or cohort) in the inclusion criteria.

**Results:**

- The authors did not comment on the quality of included studies as well as their heterogenicity.
- Figure 1:
  - The authors should clarify why 103 studies were excluded out of 131.
- The authors should perform a separate analysis (or at least a separate comment) for blood transfusion, sepsis, pleural
  effusion or visceral injury complications.  
- Table 2: the authors should add many points to describe each study adequately including:
  - Number and shape of stone
  - SFR for each study.
  - The method and timing for evaluation of SFR.
  - If SFR  was calculated after first session or after 2nd look PCNL.
  - The complications for each technique (and its rate).
  - If any procedure was Mini-PCNL and the caliber of used renal track (sheath).
  - If there were congenital renal anomalies.
  - Lithotripsy technique.
  - Operative imaging modality.

 

**Discussion:**

- The authors should give more details of the included studies.
- The authors should discuss previous meta-analyses comparing both procedures. Additionally, the authors should compare the results of the present study to previous meta-analyses clarifying any difference and any addition.

Are the rationale for, and objectives of, the Systematic Review clearly stated?

Yes

Is the statistical analysis and its interpretation appropriate?

I cannot comment. A qualified statistician is required.

Are sufficient details of the methods and analysis provided to allow replication by others?

No

Are the conclusions drawn adequately supported by the results presented in the review?

Yes

Reviewer Expertise:

Urolithiasis, endourology, PCNL, Mini-PCNL, URS, SWL

I confirm that I have read this submission and believe that I have an appropriate level of expertise to confirm that it is of an acceptable scientific standard, however I have significant reservations, as outlined above.

# Response

**Published:** 2020-08-15

**Authors:** Ponco Birowo

**Affiliations:** Department of Urology Faculty of Medicine Universitas Indonesia/ CIpto Mangunkusumo Hospital, Indonesia

**Introduction**

 Reviewers’ comment:

1. The authors reported that: [While PCNL has higher free stone rates with a similar recurrence and complication rate compared with ESWL,...]. This should be cited. Additionally, the complication rates are different between both procedures. Similarly, the recurrence rate is different.

Authors’ response:

1. Thank you very much for the comment, we have added the citation of the statement mentioned in the introduction section: [While PCNL has higher free stone rates with a similar recurrence and complication rate compared with ESWL, this procedure also has its own preparation including a guiding system, anesthesia, and positioning of the patient]. The citation was the reference number 5, which is [Ganpule AP, Vijayakumar M, Malpani A, Desai MR. Percutaneous nephrolithotomy (PCNL) a critical review. Int J Surg. 2016; 36(Pt D): 660-4.] (Page 3, Paragraph 3, Introduction section).

**Methods**

 Reviewers’ comment:

1. The authors should clarify the age of patients and the design of the study (RCT or cohort) in the inclusion criteria.

Authors’ response:

1. Thank you very much for the comment, we have revised the inclusion criteria accordingly. (Page 4, Paragraph 6, Methods section, subsection Study Selection).

**Results**

 Reviewers’ comment:

1. The authors did not comment on the quality of included studies as well as their heterogenicity.
2. Figure 1:
  - The authors should clarify why 103 studies were excluded out of 131.
3. The authors should perform a separate analysis (or at least a separate comment) for blood transfusion, sepsis, pleural effusion or visceral injury complications. 
4. Table 2: the authors should add many points to describe each study adequately including:
  - Number and shape of stone
  - SFR for each study.
  - The method and timing for evaluation of SFR.
  - If SFR was calculated after first session or after 2nd look PCNL.
  - The complications for each technique (and its rate).
  - If any procedure was Mini-PCNL and the caliber of used renal track (sheath).
  - If there were congenital renal anomalies.
  - Lithotripsy technique.
  - Operative imaging modality.

Authors’ response:

1. Thank you very much for the comment, we have added a summary of the quality assessment of each article (Page 5, Paragraph 5, Results section, subsection Study Characteristics). Furthermore, we also showed the heterogeneity of each variable measured in this article (Page 9-11, Results section, subsection Stone Free Rate, Major Complication Rate, Length of Hospital Stay, and Mean Operation Time).
2. Thank you very much for the kind comment. The reason of which 103 articles were excluded because as we screened through all the titles and abstracts, we only found 28 articles that has the main topic of our interest, which is a study with either trial design or observational study (Page 5, Paragraph 4, Results section, subsection Literature Search).
3. Thank you very much for the suggestion, we have added a further separate analysis regarding the need of blood transfusion, sepsis, and visceral injuries complication presented in a forest plot (Page 10, Results section, Figure 4).
4. Thank you very much for the suggestion, we have added more details on the Table 2 accordingly (Page 7, Results section, Table 2).

**Discussion**

 Reviewers’ comment:

1. The authors should give more details of the included studies.
2. The authors should discuss previous meta-analyses comparing both procedures. Additionally, the authors should compare the results of the present study to previous meta-analyses clarifying any difference and any addition.

Authors’ response:

1. Thank you very much for the kind comment, we have added more detail of our included studies on the discussion section (Page 11, Paragraph 3, Discussion section).
2. Thank you for the suggestion, we have added the comparison of the previous meta-analyses to our study (Page 12, Paragraph 6, Discussion section). Regarding to this addition, we have added the references accordingly (Page 15, References number 24 and 25).

# Peer review: Reviewer response for version 1

**DOI:** 10.5256/f1000research.25326.r61982

**Published:** 2020-04-06

**Authors:** Noor Buchholz, Andreas Bourdoumis

**Affiliations:** 1 U-merge Ltd. (Urology for Emerging Countries), London, UK; 2 Pennine Acute Hospitals NHS Trust, Manchester, UK

The authors need to be congratulated for this effort. They produced a comprehensive review on supine versus prone position in PCNL. This former method has been around for now almost 15 years, and still surgeons remain unsure whether there is a benefit in adopting the supine position over the established and older prone position. In that sense, this article may be a good decision tool. 

 The authors systematically reviewed the relevant literature. The reviewer misses some evidence but the authors used stringent selection criteria which may have led to their exclusion but in turn make the data more robust. An example would be:

 Kachrilas S, Papatsoris A, Bach C, Kontos S, Faruquz Z, Goyal A, Masood J,BUCHHOLZ N. Colon perforation during percutaneous renal surgery: a 10-year experience in a single endourology centre. Urol Res. 2012 Jun;40(3):263-8 ^1^.

 This article shows on a large number of patients that there is no difference in complications in the right hands. 

 Although in most experienced hands, the differences between the two methods are minimal in all aspects, and the advantages of supine are evident to most surgeons who use it, somewhat surprisingly to me the analysed data show better safety in supine, and better stone-free rate in prone PCNL. Since there seems to be no flaw in their analysis, we will have to believe this data. 

 That makes it even more important to index.

Are the rationale for, and objectives of, the Systematic Review clearly stated?

Yes

Is the statistical analysis and its interpretation appropriate?

Yes

Are sufficient details of the methods and analysis provided to allow replication by others?

Yes

Are the conclusions drawn adequately supported by the results presented in the review?

Yes

Reviewer Expertise:

Stone surgery, PCNL

We confirm that we have read this submission and believe that we have an appropriate level of expertise to confirm that it is of an acceptable scientific standard.

## References

[1] : Colon perforation during percutaneous renal surgery: a 10-year experience in a single endourology centre. *Urol Res*.2012;40(3) : 10.1007/s00240-012-0464-4 263-8 10.1007/s00240-012-0464-4 22307366
