# Supplementary material for: Supine versus prone position in percutaneous nephrolithotomy: a systematic review and meta-analysis
Source: F1000Res. 2020 Apr 2;9:231. [Version 1] doi: 10.12688/f1000research.22940.1 (PMC7509599; doi:10.12688/f1000research.22940.1)
Supplement: Peer review history

# Peer review: Reviewer response for version 1

**DOI:** 10.5256/f1000research.25326.r64021

**Published:** 2021-12-13

**Authors:** Doddy Soebadi

**Affiliations:** 1 Department of Urology, Faculty of Medicine, Airlangga University, Surabaya, Indonesia

A good review. We do need the evidence based for choosing the method.

 The authors could also state their opinions concerning the training of the procedures, whether the two methods have to be taught or directly to one method.

 In conclusion, it can also be stated the positive and negative sides of each procedures, in consideration the needs for training.

 Congratulations to the authors.

Are the rationale for, and objectives of, the Systematic Review clearly stated?

Yes

Is the statistical analysis and its interpretation appropriate?

Yes

Are sufficient details of the methods and analysis provided to allow replication by others?

Yes

Are the conclusions drawn adequately supported by the results presented in the review?

Yes

Reviewer Expertise:

Stone disease, male infertility, andrology.

I confirm that I have read this submission and believe that I have an appropriate level of expertise to confirm that it is of an acceptable scientific standard.
